# The Performance Analysis of Grouting Repair Effect on the Accuracy of Disturbance Stress Test in Damaged Surrounding Rock Mass

**DOI:** 10.3390/ma17081926

**Published:** 2024-04-22

**Authors:** Minzong Zheng, Shaojun Li, Yandu Lu, Xingan Lu, Liu Liu

**Affiliations:** 1 State Key Laboratory of Geomechanics and Geotechnical Engineering, Institute of Rock and Soil Mechanics, Chinese Academy of Sciences, Wuhan 430071, China; mzzheng@mail.whrsm.ac.cn; 2 China Water Resources and Hydropower 14th Engineering Bureau Co., Ltd., Kunming 650041, China; 18829289692@163.com (Y.L.); 18202748010@163.com (X.L.)

**Keywords:** hard rock, disturbance stress test, grouting repair, rock damage, deep tunnel

## Abstract

Disturbance stress assessment is crucial for ensuring the safety of deep engineering projects. Currently, the primary technique for continuously monitoring three-dimensional disturbance stress is the stress relief method, but its accuracy can be compromised by rock damage that occurs after excavation. To mitigate this issue, grouting is employed to repair damaged rock masses and enhance their mechanical properties. However, the impact of grouting techniques on improving the accuracy of disturbance stress testing is challenging to evaluate through laboratory and in situ experiments. To address this problem, numerical simulation technology is employed to investigate disturbance stress testing after the repair of damaged surrounding rock through grouting. The simulation results indicate that grouting repair significantly enhances the accuracy of stress testing. As the depth of damaged rock mass repair increases, the error in stress testing decreases. Achieving complete repair of the initial damage zone during grouting is essential to eliminate errors in stress testing. Expanding on the positive effects of grouting repair on stress testing, a segmented testing method for disturbance stress is proposed. The method involves separately testing the initial stress and stress changes, thereby reducing the stress level within the rock, minimizing rock failure, and enhancing the accuracy of disturbance stress testing. This study provides valuable reference methods, and the outcomes of this research will serve as a foundation for enhancing the accuracy of disturbance stress testing in deep hard rock engineering.

## 1. Introduction

In situ stress plays a crucial role in ensuring the safety and stability of deep engineering projects [1,2]. Upon excavation of tunnel projects, the initial in situ stress within the surrounding rock undergoes redistribution, resulting in disturbance stress, which can lead to rock damage and various stress-controlled failures [3,4]. Stress-induced failures, such as rib spalling, rock bursts, and stress-controlled collapse, have the potential to cause significant engineering disasters in severe cases [5,6,7]. Monitoring the variation of disturbance stress provides an effective means to evaluate the long-term behavior of hard rock in deep tunnels [8]. This understanding helps predict the level of engineering hazards and evaluate the suitability of excavation and support design schemes [9,10].

In situ stress measurement serves as an effective method for estimating disturbance stress [3,8]. Most of the techniques used to measure initial stress are also applicable to disturbance stress measurement [11,12]. Among these methods, the stress relief method is unique in its ability to continuously monitor three-dimensional stress changes [13]. It relies on linear elasticity theory and allows for the measurement of absolute stress or stress changes, depending on the specific application [14,15]. However, when monitoring stress change in deep hard rock engineering, rock damage near the tunnel wall violates the elastic assumption inherent in the stress relief method. As a result, this violation introduces significant errors in the test results [16,17,18]. Nevertheless, understanding the evolution of disturbance stress near the tunnel is of utmost importance to engineers [19,20,21]. Consequently, improving the measurement accuracy of disturbance stress in damaged surrounding rock remains a crucial challenge.

Two methods exist for testing disturbance stress using the stress relief method: one involves measuring the absolute value of disturbance stress at the measurement point using the overcoring technique, while the other method entails monitoring stress changes by embedding sensors at the measurement point and subsequently adding them to the original rock stress to derive disturbance stress [8]. Studies have demonstrated that the error in stress testing escalates with the depth of surrounding rock damage [22]. In deep hard rock engineering, the depth of surrounding rock damage correlates with the magnitude of stress, potentially resulting in greater estimation errors when measuring the absolute value of disturbance stress compared to monitoring stress changes. Consequently, separating stress changes from original rock stress testing may offer an effective approach for high geostress testing in deep engineering contexts. However, currently, no pertinent research has been identified.

On the other hand, in the context of disturbance stress monitoring, grouting is commonly employed to connect the sensor with the surrounding rock while simultaneously repairing the damaged rock [23]. In engineering practice, grouting also serves as a significant method in engineering practice for repairing fractured rock mass, effectively enhancing the strength and stiffness of the surrounding rock through filling and cementing processes [24,25,26]. However, it remains unknown whether grouting repair of the surrounding rock can effectively improve the accuracy of stress testing. The impact of grouting techniques on improving the accuracy of disturbance stress testing is challenging to evaluate through laboratory and in situ experiments.

This study aims to establish a simulation test method for disturbance stress and subsequently investigate the impact of different degrees of rock damage and grouting repair depths on the measurement accuracy of disturbance stress. Ultimately, considering the influence of grouting repair on stress testing accuracy in damaged rock, a stress subsection test (SST) method suitable for deep hard rock engineering is proposed. This method involves measuring the initial stress and stress changes separately, reducing the disturbance stress level, and minimizing rock damage through grouting, thereby improving the accuracy of disturbance stress testing. The research outcomes will serve as a reference for enhancing the measurement accuracy of disturbance stress in deep hard rock engineering.

## 2. Disturbance Stress Simulation Test in Damaged Hard Rock

### 2.1. Numerical Models and Simulation Procedures

To investigate the impact of rock damage on the accuracy of disturbance stress testing, this study uses FLAC3D 6.0 numerical software to simulate the testing process in engineering practice [27]. The stress test based on the stress relief method theory requires calculating stress by strain. When constructing a numerical model for tunnels and boreholes, it becomes necessary to mesh-encrypt the local borehole model. However, a significant size discrepancy between the tunnel and the borehole results in an excessively large grid volume, making simulation impractical.

**Figure 1:**
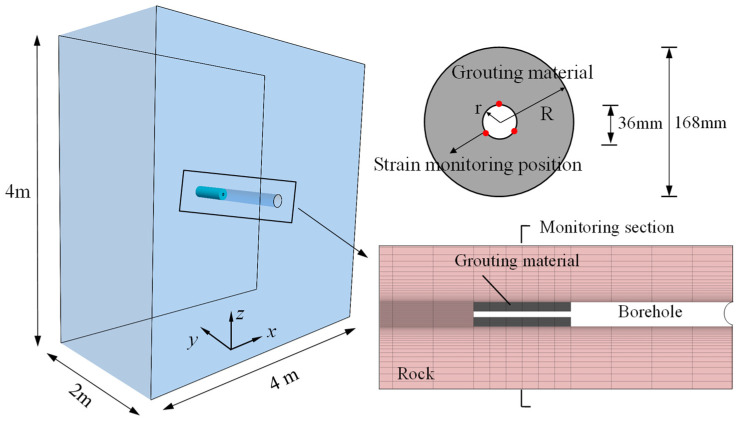
Numerical model of disturbance stress test.

To streamline the simulation process, the model consists only of boreholes, while the stress changes resulting from tunnel excavation are simplified by applying disturbance stress loading, as depicted in Figure 1. The dimensions of the model are 4 m in the x-direction, 2 m in the y-direction, and 4 m in the z-direction. To calculate the stress using the double-layer medium calculation model described in Section 2.2, the strain gauge is assumed to be affixed to the wall within the grouting body. The test hole has a diameter of 36 mm, while the grouting body has a diameter of 168 mm [28]. The y-axis corresponds to the axial direction of the borehole, and the stress testing area spans from *y* = 1.0 m to 1.5 m, covering a length of 0.5 m. The rock material is modeled as elastic–plastic material to simulate the damage occurring in deep hard rock [29].

The disturbance stress test consists of the following steps:

(a) Model establishment: The model is created, and the material parameters and initial stress conditions of the surrounding rock are assigned.
(b) Borehole drilling: A borehole is drilled to the depth of *y* = 1.5 m, indicating that the drilling has reached the desired testing position.
(c) Sensor installation: The displacement and velocity of the model are reset to zero, representing the initial state of the sensor upon installation into the borehole.
(d) Grouting body activation: The grouting body at *y* = 1.0–1.5 m is activated, and the material parameters for the grouting body are assigned.
(e) Loading disturbance stress: Disturbance stress conditions are applied at the boundaries of the model, and the equilibrium is solved.
(f) Stress calculation: The strain at the monitoring point is extracted, and the stress is calculated using the stress calculation method in the next section.

### 2.2. Disturbance Stress Simulation Test Method

**Figure 2:**
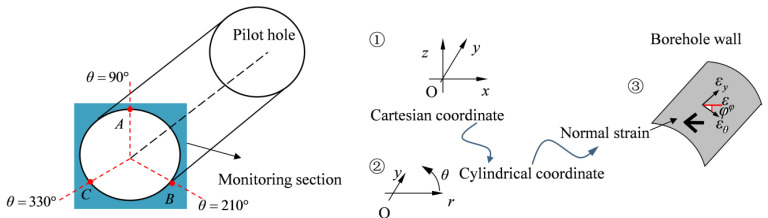
Schematic diagram of normal strain calculation of borehole wall.

To monitor the strain changes in the test holes, three monitoring points labeled A, B, and C are positioned at 120° intervals within the middle of the model at *y* = 1.25 m, as illustrated in Figure 2.

Subsequently, the strain increment in the Cartesian coordinate system (o-*xyz*) is converted to the cylindrical coordinate system (*o-ryθ*) according to Equation (1) [8]:

(1) $$\begin{array}{l} \varepsilon_{\theta }=\sin^{2}\theta \cdot \varepsilon_{x}-\sin2\theta \cdot \varepsilon_{xz}+\cos^{2}\theta \cdot \varepsilon_{z}\\ \varepsilon_{\theta y}=\sin\theta \cdot \varepsilon_{xy}-\cos\theta \cdot \varepsilon_{yz} \end{array},$$

where *θ* is the angle of the monitoring points. For points A, B, and C, they are 90°, 330°, and 210°, respectively.

(2) $$\varepsilon_{\varphi }=\sin^{2}\varphi \cdot \varepsilon_{y}+\sin2\varphi \cdot \varepsilon_{\theta y}+\cos^{2}\varphi \cdot \varepsilon_{\theta },$$

Subsequently, the normal strain in three directions (*φ* = 90°, 45°, 0°) is calculated using Equation (2), where *φ* is the angle between the testing and circumferential direction. The simulated strain increments of the borehole wall in nine directions are obtained and presented in Table 1.

In the application of stress relief test method, the calculation of stress in the double-layer medium, including rock and grout, requires assuming that the two layers are bonded together and cannot be separated. In practical engineering, the connection between two different materials may pose problems and lead to errors in stress estimation. However, the purpose of this paper is to study the promoting effect of grouting on the accuracy of disturbed stress testing in damaged surrounding rock. Considering the potential interference caused by the consideration of a non-tightly connected double-layer medium, we simplified the grouting effect, assuming that the two layers of medium can be tightly connected after grouting.

In the stress relief test method application, calculating stress in a double-layer medium comprising rock and grout necessitates the assumption that the two layers are tightly bonded and inseparable. In practical engineering, the connection between disparate materials may present challenges and introduce inaccuracies in stress estimation. Acknowledging the potential interference from a non-tightly connected double-layer medium, the grouting effect is simplified by assuming that the two layers of the medium can tightly connect after grouting. Thus, for a model containing double layers of elastic media, the equation between stress and strain is as follows [30]:

(3) $$E_{R}\varepsilon_{k}=A_{k1}\sigma_{x}+A_{k2}\sigma_{y}+A_{k3}\sigma_{z}+A_{k4}\tau_{xy}+A_{k5}\tau_{yz}+A_{k6}\tau_{zx}\begin{array}{cc} & k=1\sim 9 \end{array},$$

where

(4) $$\begin{array}{l} A_{k1}=\left[K_{1}+{µ}_{R}-2\left(1-{{µ}_{R}}^{2}\right)K_{2}\cos2\theta \right]\sin^{2}\varphi -{µ}_{R}\\ A_{k2}=\left[K_{1}+{µ}_{R}+2\left(1-{{µ}_{R}}^{2}\right)K_{2}\cos2\theta \right]\sin^{2}\varphi -{µ}_{R}\\ A_{k3}=1-(1+{µ}_{R}K_{4})\sin^{2}\varphi \\ A_{k4}=-4\left(1-{{µ}_{R}}^{2}\right)K_{2}\sin2\theta \sin^{2}\varphi \\ A_{k5}=2(1+{µ}_{R})K_{3}\cos\theta \sin2\varphi \\ A_{k6}=-2(1+{µ}_{R})K_{3}\sin\theta \sin2\varphi \end{array},$$

where $\varepsilon_{k}$ are the simulated normal strains at different orientations, $E_{R}$ and ${µ}_{R}$ are the elastic modulus and Poisson’s ratio of the rock, respectively, where

(5) $$\begin{array}{l} K_{1}=d_{1}\left(1-{µ}_{R}{µ}_{c}\right)(1-2{µ}_{c}+r^{2}/\rho^{2})+{µ}_{R}{µ}_{c}\\ K_{2}=\left(1-{µ}_{R}\right)d_{2}r^{2}+d_{3}+d_{4}{µ}_{c}/r^{2}+d_{5}/r^{4}\\ K_{3}=d_{6}\left(1+r^{2}/\rho^{2}\right)\\ K_{4}=-\left(1-2{µ}_{c}+r^{2}/\rho^{2}\right)\left({µ}_{c}-{µ}_{R}\right)\left(1-2{µ}_{c}+r^{2}/\rho^{2}\right)d_{1}/{µ}_{R}+{µ}_{c}/{µ}_{R} \end{array},$$

where

(6) $$\begin{array}{l} d_{1}=1/\left[1-2{µ}_{c}+m^{2}+\xi (1-m^{2})\right]\\ d_{2}=12\left(1-\xi \right)m^{2}(1-m^{2})/r^{2}D\\ d_{3}=\left[m^{4}(4m^{2}-3)\left(1-\xi \right)+\kappa_{1}+\xi \right]/D\\ d_{4}=-4r^{2}\left[m^{6}\left(1-\xi \right)+\kappa_{1}+\xi \right]/D\\ d_{5}=3r^{4}\left[m^{4}\left(1-\xi \right)+\kappa_{1}+\xi \right]/D\\ d_{6}=1/\left[1+m^{2}+\xi (1-m^{2})\right]\\ D=\left(1+\kappa \xi \right)\left[\kappa_{1}+\xi +\left(1-\xi \right)\left(3m^{2}-6m^{4}+4m^{6}\right)\right]\\ +\left(\kappa_{1}-\kappa \xi \right)m^{2}\left[\left(1-\xi \right)m^{6}+\kappa_{1}+\xi \right]\\ \xi =E_{c}(1+{µ}_{R})/E_{R}(1+{µ}_{c}),m=r/R,\kappa =3-4{µ}_{R},\kappa_{1}=3-4{µ}_{c} \end{array},$$

where E_c_ and μ_c_ are the elastic modulus and Poisson’s ratio of the grouting body, *r* is the radius of the testing borehole, *R* is the radius of the grouting body, and ρ is the depth of the monitoring position. The monitoring points are arranged on the surface of the test borehole in the numerical simulation; thus, ρ = r. The elastic modulus of the rock E_R_ is set as 20 GPa, Poisson’s ratio μ_R_ is set as 0.25, the elastic modulus of the grouting body E_c_ is set as 14 GPa, and Poisson’s ratio μ_c_ is set as 0.30. According to Equations (5) and (6), the K-values are calculated as K_1_ = 1.265, K_2_ = 1.165, K_3_ = 1.185, and K_4_ = 0.943, respectively. By substituting the K-values into Equation (4), and subsequently into Equation (3), the stress–strain equation can be derived to calculate the six stress components.

Finally, in order to assess the accuracy of the stress simulation test results, the Euclidean distance between the simulated test value σ_m_ and the loading value σ_d_ is obtained as the error function [31]:

(7) $$\mathrm{Error}=\sqrt{{\left(\sigma_{x}^{m}-\sigma_{x}^{d}\right)}^{2}+{\left(\sigma_{y}^{m}-\sigma_{y}^{d}\right)}^{2}+{\left(\sigma_{z}^{m}-\sigma_{z}^{d}\right)}^{2}},$$

where $\sigma_{x}^{m}$, $\sigma_{y}^{m}$, and $\sigma_{z}^{m}$ are calculated stress components by strain, and $\sigma_{x}^{d}$, $\sigma_{y}^{d}$, and $\sigma_{z}^{d}$ are the loading stress components.

## 3. Influence of Grouting Repair on Disturbance Stress Test Accuracy

### 3.1. Borehole Damage Distribution under Initial Stress and Disturbance Stress

To achieve various initial damage depths after drilling, the surrounding rock materials are defined for 13 different models, as presented in Table 2, following the stress testing simulation steps. Different depths of rock damage were formed after drilling for different models under the initial stress $\sigma_{x}^{0}$ = −30 MPa, $\sigma_{y}^{0}$ = −30 MPa, $\sigma_{z}^{0}$ = −30 MPa. It is evident that as the strength of the rock decreases, the depth of damage around the borehole progressively increases. Subsequently, the grouting body at y = 1.0–1.5 m is activated. To mitigate the influence of grouting body failure on the accuracy of stress testing, the grouting body is considered as an elastic material. Finally, disturbance stress $\sigma_{d}^{A}$ ($\sigma_{x}^{d}=-20\;MPa$, $\sigma_{y}^{d}=-20\;MPa$, $\sigma_{z}^{d}=-20\;MPa$) and $\sigma_{d}^{B}$ ($\sigma_{x}^{d}=-40\;MPa$, $\sigma_{y}^{d}=-40\;MPa$, $\sigma_{z}^{d}=-40\;MPa$) are loaded at the boundary of the model, respectively. Under the disturbance stress $\sigma_{d}^{A}$, stress change Δσ is 10 MPa, indicating tensile stress. Under the disturbance stress $\sigma_{d}^{B}$, stress change Δσ is −10 MPa, indicating compressive stress.

**Figure 3:**
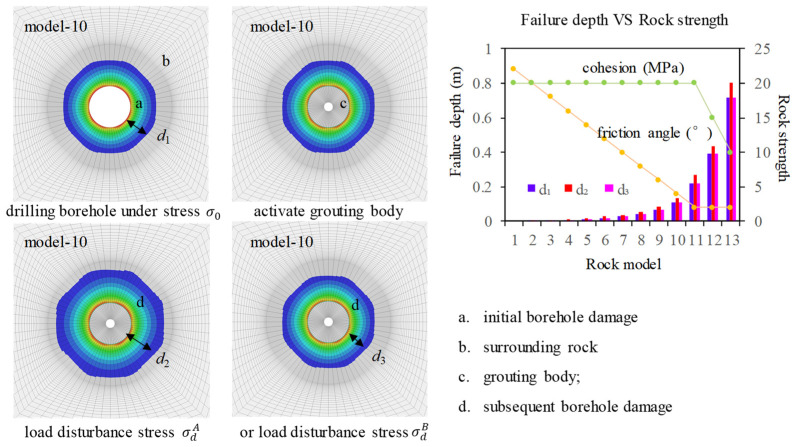
Borehole damage conditions for different rock models.

The damage resulting from the initial stress is referred to as the initial rock damage d_1_. Upon applying disturbance stress, the depth of rock damage around the borehole may further increase; this is also known as secondary damage d_2_ or d_3_. Figure 3 shows the rock failure of the borehole under initial stress σ_0_ and disturbance stress $\sigma_{d}^{A}$ and $\sigma_{d}^{B}$. It can be seen that under the compressive disturbance stress, due to the increase in hydrostatic stress, the bearing capacity of the rock increases, so the secondary damage depth of the drilling hole does not continue to increase, d_1_ = d_3_. However, under tensile disturbance stress, the bearing capacity of the rock decreases due to the decrease in hydrostatic stress; thus, the secondary damage depth of borehole d_2_ is greater than the primary damage depth d_1_. Considering model 10 as an example, under initial stress σ_0_, a failure depth of d_1_ = 0.108 m was formed around the borehole (area a in Figure 3). Under tensile disturbance stress $\sigma_{d}^{A}$, the failure depth became larger, d_2_ = 0.134 m. Meanwhile, under compressive disturbance stress $\sigma_{d}^{B}$, the damage to the rock did not increase any further.

### 3.2. Disturbance Stress Test Error under Different Borehole Damage Conditions

**Figure 4:**
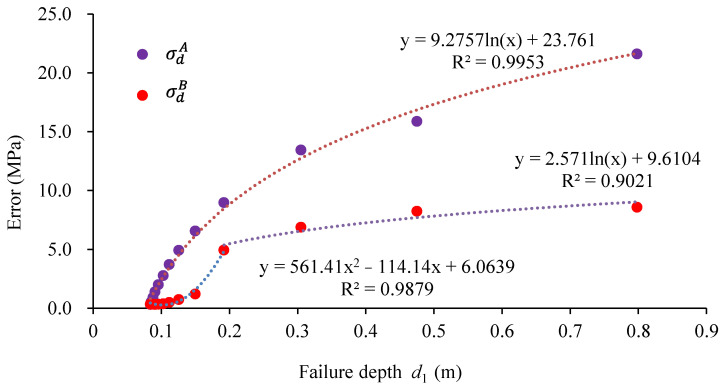
Stress test error at different initial damage depths of borehole.

The strain increments at the monitoring points were determined, and subsequently, the stress change Δσ was calculated using Equations (1)–(6). The accuracy of the stress simulation test results was calculated according to Equation (7). Figure 4 illustrates the error between the simulated test values and loading values of disturbance stress for different failure depths d_1_. As observed, when the stress change is tensile, the testing error increases logarithmically as the failure depth increases. When the stress change is compressive, the testing error remains relatively small if the failure depth of the borehole is less than 0.066 m. When the damage depth is greater than 0.108 m, the testing error increases logarithmically with an increase in the failure depth.

The numerical simulation results show that the measurement error of tensile stress disturbance stress is much larger than that of compressive stress at the same initial damage depth. In addition, the larger the borehole damage depth, the greater the testing error in stress change measurement. Thus, the accuracy of the disturbance stress test is primarily influenced by two key factors: the initial damage depth of the borehole and the tension-compressive characteristics of the disturbance stress.

### 3.3. Influence of Grouting Repair on the Disturbance Stress Test Accuracy

**Figure 5:**
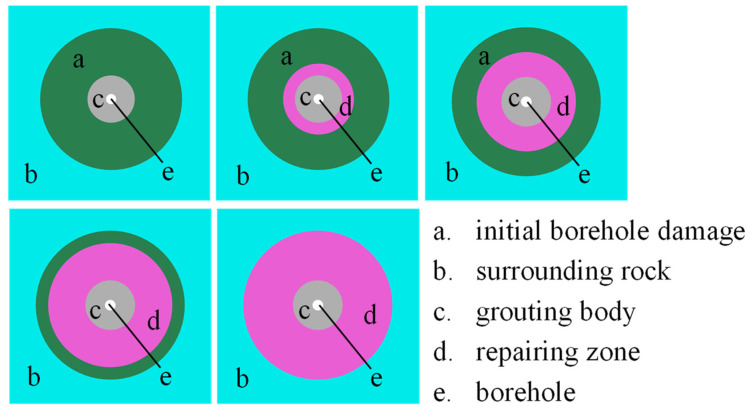
Different grouting repair depths.

The accuracy of disturbance stress testing tends to decrease as the degree of rock damage increases. Therefore, if the initial damage of the surrounding rock can be reduced through grouting repair, the accuracy of stress testing can be improved. Grouting is currently considered an important method for repairing fractured rock mass and enhancing its strength [24,25,26]. Considering the repair effect of grouting on the surrounding rock, it is assumed that after grouting is performed in the borehole (area c in Figure 3), the cement will diffuse and form a repair zone of a certain depth. In the numerical simulation, it is assumed that the repaired surrounding rock will transition from a damaged state to an elastic state to simulate the repairing effect of grouting. The depth of the surrounding rock repaired by grouting is set as 0 R; 0.5 R; and 1.0 R, 1.5 R, and 2 R, respectively, as shown in Figure 5. R is the radius of the borehole, and R = 0.084 m. The initial stress and disturbance stress loading conditions remain the same as in Section 3.1.

**Figure 6:**
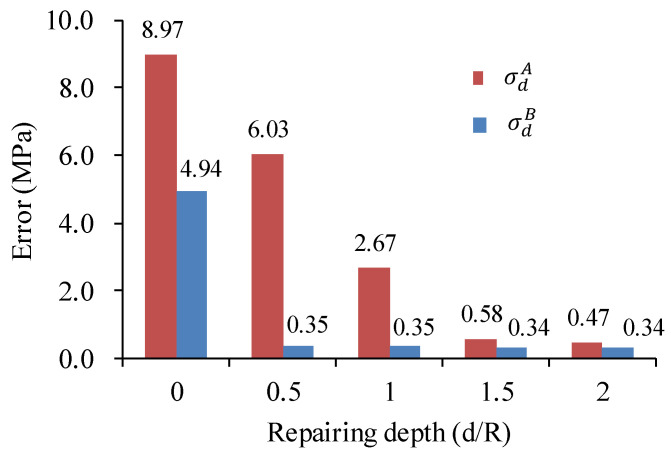
Influence of grouting repair depth on stress test accuracy.

In Figure 6, the error between the simulated test values and loading values of disturbance stress is shown under different repairing depths. It is observed that as the depth of surrounding rock repair increases, the testing error of stress gradually decreases. Under disturbance stress $\sigma_{d}^{A}$, the damage depth of the borehole increases due to the decrease in hydrostatic pressure (d_2_ = 1.6 R). When the repair depth reaches 1.5 R, the impact of the borehole damage on the stress testing error is eliminated. Under disturbance stress $\sigma_{d}^{B}$, the increase in hydrostatic pressure results in a relatively small damage depth of the borehole (d_3_ = 1.3 R). When the grouting repair depth reaches 0.5 R, which means that not all damaged areas are repaired, the stress testing error has been eliminated.

Based on the numerical simulation results, it can be concluded that if grouting is able to restore the surrounding rock from a damaged state to an elastic state, the stress testing error will decrease with the increase in the repair depth. The results indicate that after the initial damage area of the borehole is repaired, the testing error of stress can be completely eliminated. However, it should be noted that in practical engineering, grouting may not be able to fully restore the damaged surrounding rock to an elastic state. Nonetheless, it can be inferred that grouting repair will increase the strength of the rock and reduce the degree of damage to the surrounding rock. The lower the degree of rock damage, the higher the accuracy of stress testing. Therefore, the repair effect of grouting on the surrounding rock is expected to contribute to improving the accuracy of stress testing in practical engineering scenarios.

## 4. Discussion

In deep hard rock engineering construction, frequent occurrences of engineering disasters such as rock bursts and slabbing are often attributed to high geostress. Understanding the variations in disturbance stress during the construction process through stress change monitoring can provide an effective means of predicting engineering disasters. When measuring disturbance stress in deep hard rock engineering, the relaxation failure of surrounding rock can lead to a decrease in the accuracy of stress estimation. Specifically, there is a contradiction between the elastic assumptions of stress testing techniques and the non-elastic behavior of the rock mass after excavation in deep hard rock.

To address this issue, considering the repairing effect of cement grouting on damaged surrounding rock, cement slurry is used as a coupling medium to connect the rock mass and the hollow inclusion strain sensor during disturbance stress testing in deep engineering projects. Building upon this technique, this study investigates the influence of grouting repair on the accuracy of disturbance stress testing. Ultimately, considering the influence of grouting repair on stress testing accuracy in damaged rock, a stress subsection test (SST) method suitable for deep hard rock engineering is proposed. This section will introduce the SST method and discuss its effectiveness. It is important to note that this study is based on two fundamental assumptions: (1) The stress relief method requires rocks to be elastic or approximately elastic media, hence applicable to homogeneous hard, brittle rocks such as granite and marble; (2) after grouting repair, the damaged surrounding rock can restore to an elastic state. The conclusions of this study are also based on these two assumptions.

### 4.1. Analysis of Disturbance Stress in a Circular Opening

**Figure 7:**
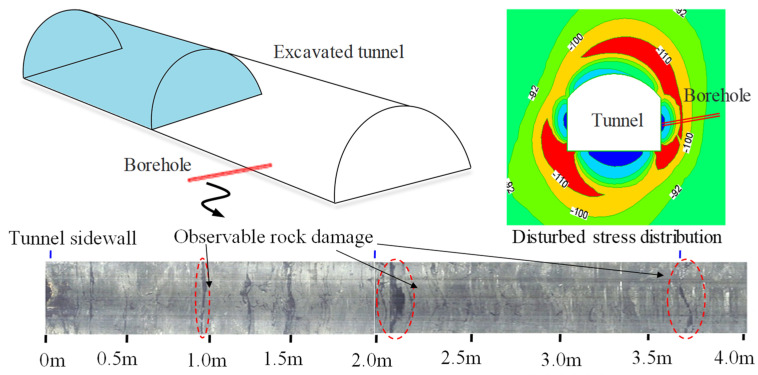
Rock damage of the borehole after tunnel excavation.

In deep, high-stress environments, excavation of underground hard rock engineering leads to the redistribution of stress around the tunnel. When the disturbance stress exceeds the strength of the rock mass, rock damage occurs around the tunnel, resulting in a relaxation zone of a certain depth, as shown in Figure 7. When testing disturbance stress within the relaxation zone, the damage to the rock violates the assumption of rock elasticity required by the stress relief method. Therefore, conducting stress tests may lead to highly inaccurate estimation results.

**Figure 8:**
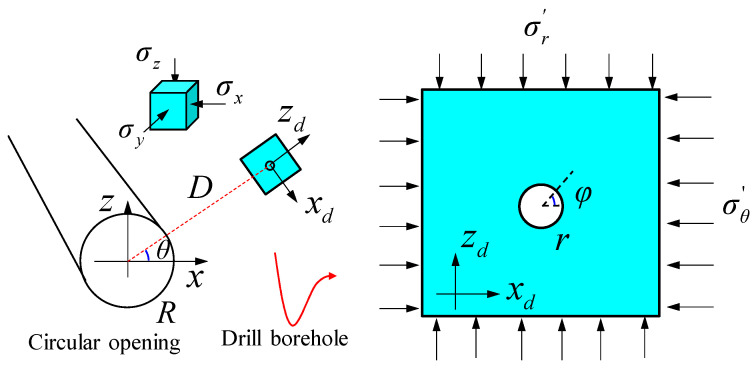
Analytical calculation model of disturbance stress in circular tunnel.

Taking a circular tunnel as an example, after excavation, the initial in situ stress within the surrounding rock transforms into secondary stresses (Fairhurst, 2003 [3]), as shown in Figure 8. The analytical solution of disturbance stress in a circular opening is provided by Fama and Pender [32]:

(8) $$\begin{array}{l} \sigma_{R}=\frac{\sigma_{x}+\sigma_{z}}{2}(1-\frac{R^{2}}{D^{2}})+\frac{\sigma_{x}-\sigma_{z}}{2}(1+\frac{3R^{4}}{D^{4}}-\frac{4R^{2}}{D^{2}})\cos2\theta \\ \sigma_{\theta }=\frac{\sigma_{x}+\sigma_{z}}{2}(1+\frac{R^{2}}{D^{2}})-\frac{\sigma_{x}-\sigma_{z}}{2}(1+\frac{3R^{4}}{D^{4}})\cos2\theta \\ \sigma_{y}=-2µ\left(\sigma_{x}-\sigma_{z}\right)\frac{R^{2}}{D^{2}}\cos2\theta +\sigma_{y} \end{array},$$

where D is the depth of the measuring point, R is the radius of the circular opening, θ is the angle between the measuring point orientation and the x-axis, and counterclockwise is positive. σ_x_, σ_y_, and σ_z_ are three initial normal stresses parallel to the coordinate axes x, y, and z, respectively. σ_R_, σ_θ_, and σ_y_ are secondary stresses along the radial, tangential, and axial directions of the tunnel, respectively.

Subsequently, a borehole with a radius of r is drilled parallel to the tunnel axis, resulting in the formation of a tertiary stress environment around the borehole. As shown in Figure 8, since the size of the borehole is relatively small compared to the tunnel, the secondary stress following tunnel excavation is approximately uniformly distributed in the vicinity of the borehole. Thus, the three normal stresses in the local coordinate axis system o-x_d_y_d_z_d_ around the borehole are as follows:

(9) $$\sigma_{x}^{d}=\sigma_{\theta },\sigma_{y}^{d}=\sigma_{y},\sigma_{z}^{d}=\sigma_{R},$$

Substituting Equation (9) into Equation (8) to obtain the tertiary stress around the borehole,

(10) $$\begin{array}{l} \sigma_{r}=\frac{\sigma_{\theta }+\sigma_{R}}{2}(1-\frac{r^{2}}{d^{2}})+\frac{\sigma_{\theta }-\sigma_{R}}{2}(1+\frac{3r^{4}}{d^{4}}-\frac{4r^{2}}{d^{2}})\cos2\varphi \\ \sigma_{\varphi }=\frac{\sigma_{\theta }+\sigma_{R}}{2}(1+\frac{r^{2}}{d^{2}})-\frac{\sigma_{\theta }-\sigma_{R}}{2}(1+\frac{3r^{4}}{d^{4}})\cos2\varphi \\ \sigma_{y_{d}}=-2µ\left(\sigma_{\theta }-\sigma_{R}\right)\frac{r^{2}}{d^{2}}\cos2\varphi +\sigma_{y} \end{array},$$

where d is the depth of the measuring points around the borehole, φ is the angle between the measuring points and the azimuth x_d_, and counterclockwise is positive. σ_r_, σ_φ_, and $\sigma_{y_{d}}$ are the radial, tangential, and axial stresses around the borehole, respectively.

According to Equation (8), the disturbance stress at the tunnel wall under the initial stress environment is (D = R):

(11) $$\begin{array}{l} \sigma_{R}=0\\ \sigma_{\theta }=\sigma_{x}\left(1-2\cos2\theta \right)+\sigma_{z}\left(1+2\cos2\theta \right)\\ \sigma_{y}=-2µ\left(\sigma_{x}-\sigma_{z}\right)\cos2\theta +\sigma_{y} \end{array},$$

According to Equation (10), the disturbance stress at the borehole wall under the secondary stress environment is

(12) $$\begin{array}{l} \sigma_{r}=0\\ \sigma_{\varphi }=\alpha \sigma_{x}-\beta \sigma_{z}\\ \sigma_{y_{d}}=-µ\left[\left(1-\alpha \right)\sigma_{x}+\left(1+\beta \right)\sigma_{z}\right]+\sigma_{y} \end{array},$$

where

(13) $$\begin{array}{l} \alpha =2\cos2\varphi \cos2\theta \left(1+\frac{3R^{4}}{d^{4}}\right)-2\cos2\varphi R^{2}/d^{2}-2\cos2\theta \left(1+2\cos2\varphi \right)\frac{R^{2}}{d^{2}}+1\\ \beta =2\cos2\varphi \cos2\theta \left(1+\frac{3R^{4}}{d^{4}}\right)+2\cos2\varphi R^{2}/d^{2}-2\cos2\theta \left(1+2\cos2\varphi \right)\frac{R^{2}}{d^{2}}-1 \end{array},$$

According to Equations (11) and (12), the minimum principal stress at the tunnel wall and borehole wall are σ_R_ and σ_r_, respectively. The maximum principal stress at the tunnel wall and the borehole wall are σ_θ_ and σ_φ_, respectively. If the rock failure complies with the M–C criterion [33], when the maximum principal stress σ_1_ exceeds the rock strength under a certain minimum principal stress σ_3_, the rock will be damaged.

(14) $$\sigma_{1}=\sigma_{3}\frac{1+\sin\varphi }{1-\sin\varphi }+\sigma_{c}$$

where σ_c_ is the uniaxial compressive strength of the rock, and φ is the friction angle.

**Figure 9:**
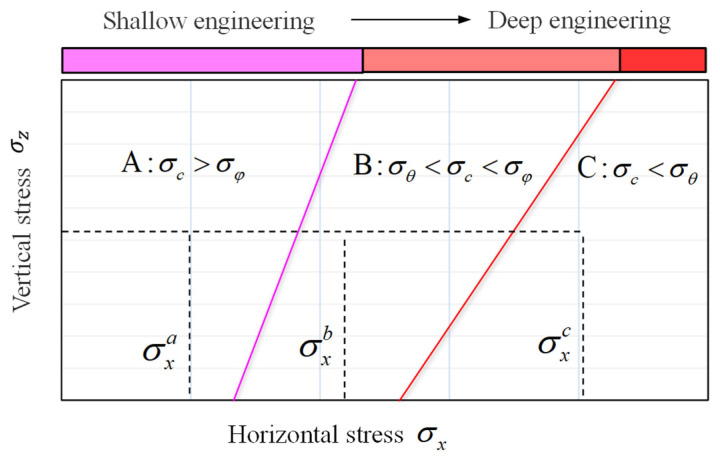
Damage conditions of boreholes in different stress environments.

According to Equations (11)–(14), the maximum principal stresses σ_φ_ and σ_θ_ at the tunnel and borehole wall under different initial stresses (σ_x_, σ_z_) are calculated and compared with the uniaxial compressive strength σ_c_. As shown in Figure 9, when σ_x_ = $\sigma_{x}^{a}$ in zone A, the maximum principal stress σ_φ_ on the borehole wall is less than the uniaxial compressive strength σ_c_. Therefore, the rock is not damaged under both initial and disturbance stress. Zone A stress prevails in shallow-buried engineering. When σ_x_ = $\sigma_{x}^{b}$ in zone B, σ_c_ < σ_φ_, the borehole wall is damaged after drilling. However, at deeper depths approaching the initial stress, the maximum principal stress on the borehole wall is close to σ_θ_ and less than σ_c_; thus, the borehole wall is not damaged. In short, rocks remain undamaged during excavation in the initial stress environment but incur damage in the disturbance stress environment. Zone B stress prevails in deep-buried engineering. When σ_x_ = $\sigma_{x}^{c}$ in zone C, σ_c_ < σ_θ_, the borehole wall is damaged under the initial stress. The stress in zone C prevails in deep-buried engineering in high-stress environments.

### 4.2. SST Method for Disturbance Stress Testing under High-Stress Environments

The stress relief method, including the overcoring technique, is commonly used to measure disturbance stress in deep rock engineering. The analytical solution reveals that rock damage occurs during the measurement of disturbance stress in deep, high-stress environments, leading to inaccurate stress test results [34]. If the testing error is substantial and the method is deemed unsuccessful, the feasibility of stress measurement depends on whether damage will occur at the testing location under the current stress environment. According to the stress environment zoning in Figure 9, three scenarios can be identified: (1) In Zone A, both the initial and disturbance stress can be tested; (2) in Zone B, the initial stress can be tested while disturbance stress cannot; (3) in Zone C, neither the initial nor the disturbance stress can be tested. Therefore, to reduce the error in stress testing, the key lies in controlling the degree of rock damage.

**Figure 10:**
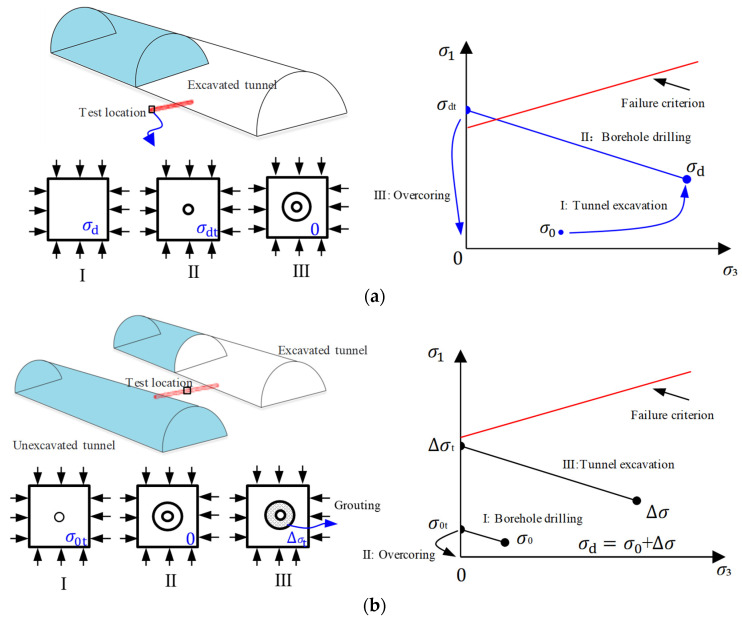
Schematic of disturbance stress testing process and stress path: (**a**) overcoring stress relief method; (**b**) stress staged test method.

For the stress environment in Zone B, the rock remains undamaged during excavation under the initial in situ stress but incurs damage under disturbance stress. Figure 10a illustrates the stress path during the measurement of disturbance stress using the overcoring stress relief method under the stress environment in Zone B. In stage Ⅰ, the initial stress σ_0_ at the testing location changes to disturbance stress σ_d_ after tunnel excavation. In stage II, when drilling the borehole at the testing location, the secondary stress σ_d_ at the borehole wall transforms into tertiary stress σ_dt_. The magnitude of the tertiary stress σ_dt_ exceeds the strength envelope of the rock, causing the rock to be damaged. Therefore, when applying the overcoring stress relief method in stage III to measure disturbance stress, the stress testing error will increase with the degree of rock damage.

To mitigate the testing error of disturbance stress, one approach is to minimize the degree of rock damage at the test location by reducing the stress level. Figure 10b presents an alternative process for disturbance stress testing called the SST method, which aims to achieve more accurate stress measurements. The method involves decomposing the disturbance stress into two components: initial stress σ_0_ and stress change Δσ for testing. The first step is to utilize the overcoring stress relief method before tunnel excavation to measure the initial stress σ_0_. This is achieved by drilling boreholes from adjacent excavated tunnels. In stage Ⅰ, the initial stress of the borehole wall transforms into secondary stress σ_0t_ after drilling. The rock remains undamaged during excavation under the initial in situ stress for the stress environment in Zone B. Therefore, the overcoring stress relief method can accurately determine the initial stress (σ_0_) in stage II. The second step involves placing a stress sensor in the borehole and coupling it with the surrounding rock through grouting. In stage III, after tunnel excavation, the stress around the grouting body becomes Δσ. Under the stress change Δσ, the secondary stress Δσ_t_ at the borehole wall remains within the elastic range and does not exceed the strength envelope. Consequently, the stress change Δσ can be accurately determined.

By adjusting the testing process of disturbance stress, this method effectively reduces the stress level at the test location. As a result, the disturbance stress no longer exceeds the strength envelope, avoiding the associated testing errors. This approach, known as the SST method, enables more accurate stress measurements by decomposing the disturbance stress into the initial stress and stress change components.

However, in the stress environment of Zone C, rock sustains damage during excavation under both initial stress and disturbance stress. Consequently, the borehole experiences initial damage, and when employing stress relief methods to test the initial stress, the testing error cannot be eliminated. However, when testing stress change, as indicated in Section 3.3, considering the reparative effect of grouting on the borehole demonstrates that the error in stress variation testing decreases with an increase in repair depth. If the initial damage zone is completely repaired, there will be no error in the results of stress variation testing. At this point, the SST method remains effective.

### 4.3. The Effect of SST Perturbation Stress Measurement Method

**Figure 11:**
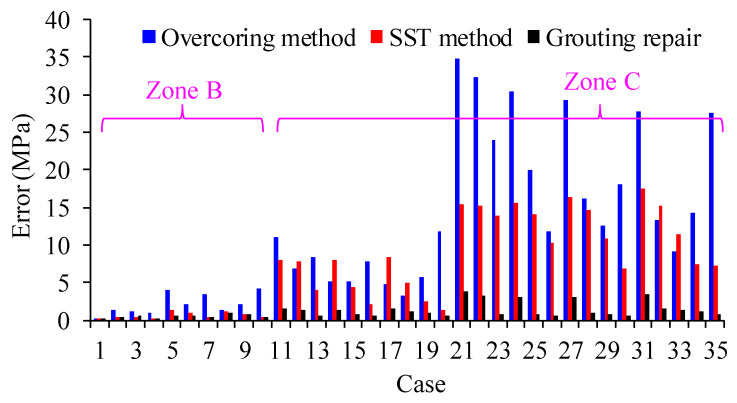
Stress test errors under different methods.

To assess the effectiveness of the SST method in improving the accuracy of disturbance stress testing in deep high-stress environments, 35 different disturbance stress conditions were conducted using both the SST method and the overcoring stress relief method, as described in Figure 10. The results are presented in Table 3, and the testing accuracy is shown in Figure 11.

In Figure 11, it can be observed that under the stress conditions in Zone B, the SST method significantly reduces testing errors compared to the overcoring stress relief method. This indicates the effectiveness of the SST method in enhancing stress testing accuracy. However, in the stress conditions of Zone C, the existence of initial damage in the borehole leads to relatively high stress testing errors. There are instances where the testing error remains significant even under the SST method, reaching as high as 16.40 MPa in case 27. By taking into account the reparative impact of grouting on the initial damage of the drilling hole, as outlined in Section 3.3, the accuracy of stress testing for significant conditions can be enhanced. Figure 11 illustrates that by taking into account the grouting repair effect, the stress testing error is completely eliminated. Therefore, repairing the initial rock damage can further eradicate testing errors and enhance the accuracy of stress testing.

## 5. Conclusions

The stress redistribution in the surrounding rock mass occurs during the excavation of deep tunnels, transitioning from initial stress to disturbance stress. Evaluating the safety of deep engineering projects relies on accurately assessing disturbance stress. Currently, the stress relief method is the sole approach for continuous monitoring of disturbance stress variations. However, the presence of rock damage during stress relief testing can compromise the reliability of results. Hence, enhancing the accuracy of testing disturbed stress in damaged surrounding rock is a crucial concern. Grouting offers a means to repair damaged rocks and enhance their mechanical properties. This study employs numerical simulation to investigate the impact of rock damage and grouting repair on the accuracy of disturbance stress testing. The key findings are as follows:

(1) Excavation or drilling in deep, high-stress environments can result in rock damage, which affects the accuracy of disturbance stress testing using the stress relief method. The testing error increases with the depth of the damaged zone.
(2) The repair effect of grouting on the surrounding rock will help improve the accuracy of stress testing, and the error of stress testing will decrease with the increase in repair depth.
(3) Taking into account the restorative properties of grouting in rock formations, a segmented testing approach for disturbance stress is introduced. This method entails conducting separate tests to measure the initial stress and stress changes, effectively lowering the internal stress levels within the rock. By reducing the extent of rock damage, this approach enhances the precision of disturbance stress testing.
(4) During the practical implementation of disturbance stress testing, it is recommended to utilize high-strength and well-repaired grouting materials. This serves a dual purpose: firstly, it facilitates the repair of initial damage within the borehole; secondly, it safeguards the grouting body against damage within the disturbance stress environment. By adhering to these guidelines, the reliability of stress testing results can be significantly enhanced.

## Figures and Tables

**Table 1:** Strain measurement position.

k	1	2	3	4	5	6	7	8	9
*θ*	90°	90°	90°	210°	210°	210°	330°	330°	330°
*φ*	90°	45°	0°	90°	45°	0°	90°	45°	0°

**Table 2:** Rock failure depth at different strength parameters.

Model	Cohesion /c (MPa)	Friction Angle /*φ* (deg)	Failure Depth under σ_0_/d_1_ (m)	Failure Depth under $\sigma_{d}^{A}$/d_2_ (m)	Failure Depth under $\sigma_{d}^{B}$/d_3_ (m)
1	22	20	0.000	0.000	0.000
2	20	20	0.001	0.002	0.001
3	18	20	0.004	0.004	0.004
4	16	20	0.007	0.013	0.007
5	14	20	0.012	0.019	0.012
6	12	20	0.019	0.027	0.019
7	10	20	0.028	0.038	0.028
8	8	20	0.042	0.056	0.042
9	6	20	0.066	0.083	0.066
10	4	20	0.108	0.134	0.108
11	2	20	0.221	0.267	0.221
12	2	15	0.391	0.433	0.391
13	2	10	0.714	0.805	0.714

**Table 3:** Stress loading conditions.

Loading Case	Initial Stress			Disturbance Stress			Stress Zoning
	$\sigma_{x}^{o}$ (MPa)	$\sigma_{y}^{o}$ (MPa)	$\sigma_{z}^{o}$ (MPa)	$\sigma_{x}^{d}$ (MPa)	$\sigma_{y}^{d}$ (MPa)	$\sigma_{z}^{d}$ (MPa)	
1	10	10	10	20	20	20	B
2	20	10	10	30	20	20	B
3	20	20	10	30	30	20	B
4	20	20	20	30	30	30	B
5	30	10	10	40	20	20	B
6	30	20	10	40	30	20	B
7	30	20	20	40	30	30	B
8	30	30	10	40	40	20	B
9	30	30	20	40	40	30	B
10	30	30	30	40	40	40	B
11	40	10	10	50	20	20	C
12	40	20	10	50	30	20	C
13	40	20	20	50	30	30	C
14	40	30	10	50	40	20	C
15	40	30	20	50	40	30	C
16	40	30	30	50	40	40	C
17	40	40	10	50	50	20	C
18	40	40	20	50	50	30	C
19	40	40	30	50	50	40	C
20	40	40	40	50	50	50	C
21	50	10	10	60	20	20	C
22	50	20	10	60	30	20	C
23	50	20	20	60	30	30	C
24	50	30	10	60	40	20	C
25	50	30	20	60	40	30	C
26	50	30	30	60	40	40	C
27	50	40	10	60	50	20	C
28	50	40	20	60	50	30	C
29	50	40	30	60	50	40	C
30	50	40	40	60	50	50	C
31	50	50	10	60	60	20	C
32	50	50	20	60	60	30	C
33	50	50	30	60	60	40	C
34	50	50	40	60	60	50	C
35	50	50	50	60	60	60	C

## Data Availability

Data are contained within the article.
